# Autofluorescence-Guided Total Thyroidectomy in Low-Volume, Nonparathyroid Institutions

**DOI:** 10.1001/jamanetworkopen.2024.11384

**Published:** 2024-05-15

**Authors:** Ali Abood, Lars Rolighed, Frédéric Triponez, Peter Vestergaard, Jacob Bach, Therese Ovesen

**Affiliations:** 1 Department of Otorhinolaryngology, Goedstrup Hospital, Herning, Denmark; 2 Department of Otorhinolaryngology, Head- and Neck Surgery, Aarhus University Hospital, Aarhus, Denmark; 3 Department of Thoracic and Endocrine Surgery, University Hospitals of Geneva, Geneva Switzerland; 4 Department of Endocrinology, Aalborg University Hospital, Aalborg, Denmark; 5 Steno Diabetes Center North Denmark, Aalborg University Hospital, Aalborg, Denmark; 6 Department of Otorhinolaryngology, Hospital South West Jutland, Esbjerg, Denmark

## Abstract

**Importance:**

Hypoparathyroidism following thyroid surgery is a serious complication that occurs frequently when surgery is performed by low-volume thyroid surgeons without experience in parathyroid surgery.

**Objective:**

To evaluate the occurrence of hypoparathyroidism following total thyroidectomy after the introduction of autofluorescence in low-volume, nonparathyroid institutions.

**Design, Setting, and Participants:**

This prospective, multicenter cohort study, with a follow-up period of up to 1 year, was conducted in Denmark at 2 low-volume nonparathyroid institutions between January 2021 and November 2023. All adult patients referred for total thyroidectomy were assessed for eligibility (n = 90). Only patients with no history of thyroid surgery were considered (n = 89). Patients who only underwent lobectomy (n = 6) or declined to participate (n = 5) were excluded. All included patients completed follow-up. The prospective cohort was compared with a historical cohort of successive patients undergoing primary total thyroidectomy from 2016 to 2020 (before autofluorescence was available).

**Intervention:**

Included patients underwent autofluorescence-guided total thyroidectomy.

**Main outcomes and Measures:**

Rate of hypoparathyroidism. Immediate hypoparathyroidism was defined as the need for active vitamin D postoperatively, whereas permanent hypoparathyroidism was considered when there still was a need for active vitamin D 1 year after surgery.

**Results:**

Seventy-eight patients underwent autofluorescence-guided surgery (mean [SD] age, 55.6 [13.1] years; 67 [86%] female) and were compared with 89 patients in the historical cohort (mean [SD] age, 49.7 [12.8] years; 78 [88%] female). The rate of immediate hypoparathyroidism decreased from 37% (95% CI, 27%-48%) to 19% (95% CI, 11%-30%) after the introduction of autofluorescence (*P* = .02). Permanent hypoparathyroidism rates decreased from 32% (95% CI, 22%-42%) to 6% (95% CI, 2%-14%) (*P* < .001), reaching 0% at the end of the study. More parathyroid glands were identified with autofluorescence (75% [95% CI, 70%-80%] vs 61% [95% CI, 56%-66%]) (*P* < .001) and less parathyroid glands were inadvertently excised (4% [95% CI, 1%-11%] vs 21% [95% CI, 13%-31%]) (*P* = .001).

**Conclusions and Relevance:**

In this cohort study of autofluorescence-guided thyroid surgery in low-volume, nonparathyroid institutions, the use of autofluorescence was associated with a significant decrease in both immediate and permanent hypoparathyroidism. When autofluorescence was used, hypoparathyroidism rates were comparable with those of high-volume surgeons who also perform parathyroid surgery.

## Key Points

**Question:**

Is the use of autofluorescence in thyroid surgery associated with lower hypoparathyroidism rates following total thyroidectomy in low-volume thyroid institutions without experience in parathyroid surgery?

**Findings:**

In this cohort study of 78 patients who underwent autofluorescence-guided total thyroidectomy and were compared with a historical cohort of 89 patients undergoing conventional total thyroidectomy, the rates of both immediate and permanent hypoparathyroidism decreased significantly after the introduction of autofluorescence.

**Meaning:**

These results suggest that low-volume institutions with no experience in parathyroid surgery may have lower rates of postoperative hypoparathyroidism when using autofluorescence in thyroid surgery.

## Teaser

This cohort study investigates the rates of hypoparathyroidism following total thyroidectomy after the introduction of autofluorescence-guided surgery in low-volume, nonparathyroid institutions in Denmark.

## Introduction

Hypoparathyroidism (hypoPT) is a common and serious complication associated with total thyroidectomy.^1,2,3^ It is well established that hypoPT following total thyroidectomy decreases the quality of life.^4,5,6,7,8,9^ However, recent studies have also shown a hitherto unknown association between permanent hypoPT and cardiovascular disease, kidney disease, and malignant disease.^10,11^ Patients with permanent hypoPT even seem to have an increased mortality.^12^ In addition, recent studies have also found that permanent hypoPT occurs with much higher frequencies than previously reported, reaching up to 36%.^13,14,15,16,17,18^ Thus, the severity and significance of this condition is potentially of much larger magnitude than previously assumed.

Postoperative hypoPT is caused by intraoperative damage to the parathyroid glands (PGs) or their vascular supply. The small nature of PGs and their visual similarities to fat and thyroid tissue creates a challenge in terms of adequate intraoperative PG identification and preservation.^19,20,21^ Especially low-volume thyroid surgeons who do not perform parathyroid surgery seem to find PG identification particularly challenging, resulting in an increased risk of PG damage and subsequent hypoPT.^13,14,17,22,23,24^

In recent years, the use of intraoperative near-infrared autofluorescence (NIRAF) during thyroid surgery has gained traction.^25,26,27,28,29,30^ Near-infrared autofluorescence refers to PGs ability to fluoresce when excited with light at the near-infrared wavelength of 785 nm.^31^ Thus, NIRAF holds the potential to facilitate intraoperative PG visualization.

The role of NIRAF in low-volume thyroid institutions that do not perform parathyroid surgery has not yet been examined. Such institutions are of particular interest, as they seem to have an increased risk of PG damage and subsequent hypoPT. In this study, we aimed to evaluate the occurrence of hypoPT following total thyroidectomy after the introduction of NIRAF in low-volume, nonparathyroid institutions.

## Methods

This prospective, multicenter cohort study was conducted at the departments of otorhinolaryngology at Goedstrup Hospital and Hospital South West Jutland in Denmark. The study was conducted in accordance with the Strengthening the Reporting of Observational Studies in Epidemiology (STROBE) reporting guideline and approved by the Ethical Research Committee at the Central Denmark Region.

Each department in the study had 4 thyroid surgeons and performed between 100 and 130 thyroid surgical procedures annually, mostly as hemithyroidectomies. Each surgeon performed less than 10 total thyroidectomies and approximately 30 hemithyroidectomies per year. Parathyroid surgery was not performed in either of the departments. Thus, they were both considered as low-volume, nonparathyroid institutions.^32,33^

Patients referred to the departments for total thyroidectomy were assessed for eligibility. Patients who previously had undergone thyroid surgery were excluded. Remaining patients were offered participation in the study. Patients had to provide both oral and written consent to be included. Included patients did not receive any stipend for participation in the study.

All included patients underwent NIRAF-guided total thyroidectomy (NIRAF group) from January 2021 to September 2023. The primary outcome was assessed at the end of follow-up in November 2023 and was defined as the rate of postoperative hypoPT (both immediate and permanent) compared with a historical cohort of successive patients undergoing primary total thyroidectomy between 2016 and 2020 before NIRAF was used (control group).^13^ The control group was identified through the procedure codes for total thyroidectomy (NOMESCO Classification of Surgical Procedures: KBAA25 and KBAA60). Secondary outcomes were PG identification rates, rates of autotransplantation, and inadvertent PG excision.

### Definitions

Immediate hypoPT was considered when hypocalcemia requiring treatment with active vitamin D was present postoperatively. Permanent hypoPT was defined as a condition where parathyroid hormone (PTH) secretion was insufficient to maintain normal plasma calcium levels without treatment with active vitamin D 1 year following surgery.^34^ In practice, this meant that all patients who received active vitamin D postoperatively were considered as having immediate hypoparathyroidism, whereas patients who still received active vitamin D 1 year following surgery were considered as having permanent hypoparathyroidism. One year was used as a cutoff instead of 6 months due to the possibility for late PG recovery beyond 6 months post surgery.^35^ Patients who solely received calcium supplementations postoperatively were not considered to have hypoparathyroidism as calcium and cholecalciferol supplementations are widely used in Denmark, even by healthy individuals, due to the limited sun exposure. Normocalcemia was defined as plasma ionized calcium concentrations in the normal range (1.18-1.32 mmol/L). The normal range for plasma PTH was 1.6 to 6.9 pmol/L.

### Surgical Procedures

All surgical procedures in the NIRAF group were performed using the NIRAF-based device Fluobeam LX (Fluoptics). Once activated, the NIRAF probe had to be pointed toward the surgical field for PG visualization. A PG would appear as a glowing subject on the adjacent Fluobeam LX screen. NIRAF was used at several steps during surgery, including on the removed specimen before sending it for histological assessment. For each surgical procedure in the NIRAF group, the surgeons had to fill out a specific form, providing details about PG identification and management. In each case, the surgeons had to state the number of PGs identified and the number of autotransplanted PGs. Furthermore, the surgeons also had to state how many times NIRAF was used during surgery, when NIRAF was used, and the number of PGs identified with NIRAF before the naked eye. Lastly, the surgeons had to state if they found NIRAF useful in that particular procedure. Nerve monitoring was used routinely, and magnifying loupes were used upon surgeons’ request.

### Follow-Up

Plasma levels of PTH, ionized calcium (Ca^2+^), 25-hydroxyvitamin D (D2 and D3), thyroid stimulating hormone (TSH), and creatinine were measured at baseline. Plasma parathyroid hormone and Ca^2+^ were assessed again on postoperative day 1 (POD1) and at a minimum 2 months postoperatively (first follow-up). For patients with normal parathyroid function at that point, no further follow-up was planned. Patients who still fulfilled the criteria for hypoPT at first follow-up were followed up until 1 year following surgery (second follow-up). If hypoPT still was present at that point, patients were considered as having permanent hypoPT, and follow-up related to this study was terminated. Recurrent laryngeal nerve function was assessed by flexible laryngoscopy on POD1 in each case.

### Statistical Analysis

Based on a power of 80%, a level of significance of 5%, and an anticipated reduction in the risk of permanent hypoPT from 14% in the background population^36^ to 4%, an initial sample size of 71 patients was defined. Accounting for a potential loss to follow-up of 10%, the final sample size was determined to be a total of 78 patients.

Continuous data were presented as means with SDs or medians with IQRs, depending on the distribution. Normally distributed data with equal variance were tested by the Student *t* test, while data with unequal variance were tested by the Welch test. Right-shifted data were converted logarithmically prior to comparative testing. Data that were not normally distributed were tested nonparametrically by the Mann-Whitney *U* test. Binary data were compared using Fisher exact test or the χ^2^ test. Adjustment for potential confounders was performed using a binary regression model. Potential associations were evaluated by linear regression with calculation of relevant slopes (α) with 95% CIs and *P* values. A 2-tailed *P* < .05 was considered statistically significant. Stata version 18.0 SE (StataCorp) was used for data analysis from November to December 2023.

## Results

**Figure 1.**
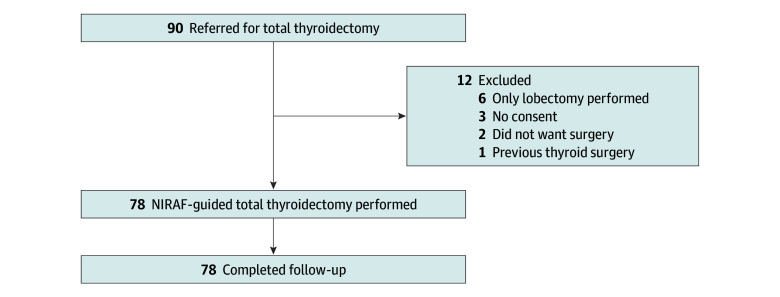
Flowchart of Patient Inclusion NIRAF indicates near-infrared autofluorescence.

A total of 167 patients were included in the final analysis. Ninety patients were initially assessed for eligibility for NIRAF-guided surgery. Seven patients did not meet the inclusion criteria, and 5 patients declined to participate (Figure 1). Finally, 78 patients underwent NIRAF-guided total thyroidectomy (mean [SD] age, 55.6 (13.1) years; 67 [86%] female) performed by 8 different surgeons. The control group consisted of 89 patients who were identified in the historical cohort where NIRAF was not used (mean [SD] age, 49.7 [12.8] years; 78 [88%] female).^13^ Surgical procedures in the historical cohort were performed by a total of 6 surgeons. Three of the surgeons performed surgical procedures in both the NIRAF group and the control group. All surgeons in both groups had an annual volume of less than 10 total thyroidectomies, and none of the surgeons performed parathyroid surgery. Patients were slightly older in the NIRAF group than in the control group (mean [SD] age, 55.6 [13.1] years vs 49.7 years [12.8] years; *P* = .004). Otherwise, the 2 groups did not differ at baseline (Table 1).

**Table 1.** Baseline Characteristics of Included Patients

Characteristic	Patients, No. (%)		*P* value
	NIRAF (n = 78)	Controls^a^ (n = 89)	
Age, mean (SD), y	55.6 (13.1)	49.7 (12.8)	.004^b^
Sex			
Female	67 (86)	78 (88)	.82^c^
Male	11 (14)	11 (12)	
BMI, mean (SD)	28.6 (5.6)	29.1 (6.4)	.73^c^
Indication for surgery			
Goiter	42 (54)	38 (43)	.17^c^
Graves disease	30 (39)	37 (42)	.75^c^
Thyrotoxicosis	6 (8)	14 (16)	.15^c^
Biochemistry			
P-PTH, median (IQR), pmol/L	6 (4.6-8.2)	7 (5.1-8.5)	.26^d^
P-Ca^2+^, mean (SD), mmol/L	1.25 (0.04)	1.26 (0.04)	.73^b^
P-25-hydroxy-vitamin D (D2+D3), median (IQR), nmol/L	72 (52-92)	72 (59-95)	.62^d^
P-TSH, median (IQR), 10^−3^ IE/L	0.671 (0.247-1.335)	0.567 (0.019-1.145)	.17^d^
P-creatinine, median (IQR), μmol/L	65 (57-71)	63 (56-74)	.93^d^

Abbreviations: BMI, body mass index; NIRAF, near-infrared autofluorescence; P-Ca^2+^, plasma ionized calcium; P-creatinine, plasma creatinine; P-PTH, plasma parathyroid hormone; P-TSH, plasma thyroid stimulating hormone.

SI conversion factors: To convert P-Ca^2+^ to mg/dL, divide by 0.25; P-creatinine to mg/dL, divide by 88.4; to convert P-PTH to pg/mL, multiply by 9.425; P-25-hydroxy-vitamin D to ng/mL, divide by 2.496.

^a^ Abood et al,^13^ 2024.

^b^ Analyzed using Student *t* test.

^c^ Analyzed using Fisher exact test.

^d^ Analyzed using the Mann-Whitney *U* test.

The rate of immediate hypoPT before the use of NIRAF was 37% (95% CI, 27%-48%). After the introduction of NIRAF, the rate of immediate hypoPT decreased to 19% (95% CI, 11%-30%) (*P* = .02). Likewise, the rate of permanent hypoPT decreased significantly from 32% (95% CI, 22%-42%) to 6% (95% CI, 2%-14%) (*P* < .001) after the introduction of NIRAF (Table 2). The age-adjusted relative risk (RR) for hypoPT when NIRAF was used was 0.49 (95% CI, 0.28-0.83) (*P* = .009) for immediate hypoPT, and 0.20 (95% CI: 0.08–0.50, *P* = .001) for permanent hypoPT (Table 3). The proportion of patients with plasma PTH levels below the normal range at final follow-up was 3% (95% CI, 0%-9%) in the NIRAF group and 17% (95% CI, 10%-26%) in the control group (*P* = .002).

**Table 2.** Comparison of Outcomes Between the NIRAF Group and the Control Group

Outcome	Mean (SD)		*P* value
	NIRAF (n = 78)	Controls^a^ (n = 89)	
Surgery-related outcomes			
No. of PGs identified (%)	234 (75)	218 (61)	<.001^b^
PG autotransplantation, No. of patients (%)	20 (26)	17 (19)	.35^b^
PG autotransplantation, No. of PGs	31 (10)	18 (5)	.007^b^
Weight of specimen, median (IQR)	88 (56-152)	96 (45-181)	.49^c^
Duration of surgery, min	137 (30)	116 (28)	<.001^d^
Duration of hospitalization, median (IQR), days	2 (1-3)	3 (2-4)	<.001^c^
Autofluorescence, No. (%)			
No. of times autofluorescence was used	7 (2)	NA	NA
No. of PGs identified with NIRAF before the naked eye	148 (63)	NA	NA
Timing of NIRAF application during surgery			
Before dissection of upper thyroid pole	53 (68)	NA	NA
Before dissection of lower thyroid pole	67 (86)	NA	NA
Before latero-posterior dissection	61 (78)	NA	NA
On the removed specimen	70 (90)	NA	NA
Benefit of using NIRAF, No. (%)			
No benefit	4 (5)	NA	NA
Some benefit	25 (32)	NA	NA
Great benefit	48 (62)	NA	NA
Not reported	1 (1)	NA	NA
Complications, No. (%)			
Immediate RLN injury	3 (2)	11 (6)	.06^b^
Permanent RLN injury	0	5 (3)	.06^b^
Postoperative bleeding	3 (4)	4 (5)	>.99^b^
Postoperative infection	2 (3)	3 (3)	>.99^b^
Biochemistry			
P-PTH POD1, median (IQR), pmol/L	3.4 (1.5-4.4)	1.4 (.5-3.4)	<.001^c^
P-Ca^2+^ POD1	1.19 (0.07)	1.15 (0.07)	.002^d^
P-PTH POD1 < 1.6 pmol/L, No. (%)	20 (26)	45 (49)	.001^b^
P-Ca^2+^ POD1 < 1.18 mmol/L, No. (%)	27 (35)	57 (64)	<.001^b^
P-PTH, First follow-up, median (IQR), pmol/L	4.8 (3.7-6.4)	3.6 (2.1-5.4)	<.001^c^
P-Ca^2+^ First follow-up, mmol/L	1.23 (0.05)	1.20 (0.06)	<.001^e^
P-PTH First follow-up <1.6 pmol/L, No. (%)	2 (3)	14 (6)	.004^b^
P-Ca^2+^ First follow-up <1.18 mmol/L, No. (%)	7 (9.0)	19 (21)	.03^b^
P-PTH Second follow-up <1.6 pmol/L, No. (%)	2 (3)	15 (17)	.002^b^
P-Ca^2+^ Second follow-up <1.18 mmol/L, No. (%)	5 (6)	21 (24)	.003^b^
Histology, No. (%)			
Benign nodules	40 (51)	52 (58)	.44^b^
Hyperplasia	26 (33)	29 (33)	>.99^b^
Inflammation	5 (6)	4 (5)	.74^b^
Riedels thyroiditis	0	1 (1)	>.99^b^
Malignant disease	7 (9)	4 (5)	.35^b^
Inadvertently excised PGs	4 (1)	23 (7)	<.001^b^
Inadvertent PG excision, No. of patients	3 (4)	19 (21)	.001^b^
HypoPT, No. (%)			
Immediate	15 (19)	33 (37)	.02^b^
Permanent	5 (6)	28 (32)	<.001^b^

Abbreviations: HypoPT, hypoparathyroidism; NIRAF, near-infrared autofluorescence; P-Ca^2+^, plasma ionized calcium; PG, parathyroid gland; POD1, postoperative day 1; P-PTH, plasma parathyroid hormone; RLN, recurrent laryngeal nerve.

SI conversion factors: To convert P-Ca^2+^ to mg/dL, divide by 0.25; P-PTH to pg/mL, multiply by 9.425.

^a^ Abood et al,^13^ 2024.

^b^ Analyzed using Fisher exact test.

^c^ Analyzed using the Mann-Whitney *U* test.

^d^ Analyzed using Student *t* test.

^e^ Analyzed using Welch test; 1st follow-up: minimum 2 months postoperatively; 2nd follow-up: 1 year postoperatively.

**Table 3.** Relative Risks for Primary and Secondary Outcomes With NIRAF vs Without NIRAF

	Crude RR (95% CI)^a^	*P* value	Age-adjusted RR (95% CI)^b^	*P* value
Immediate hypoPT	0.52 (0.31-0.88)	.01	0.49 (0.28-0.83)	.009
Permanent hypoPT	0.20 (0.08-0.50)	<.001	0.20 (0.08-0.50)	.001
PG identification rate	1.22 (1.10-1.36)	<.001	1.23 (1.11-1.37)	<.001
PG autotransplantation, No. of patients	1.34 (0.76-2.38)	.31	1.47 (0.82-2.63)	.19
PG autotransplantation, No. of PGs	1.97 (1.12-3.44)	.02	2.30 (1.30-4.06)	.004
Inadvertent PG excision, No. of patients	0.18 (0.06-0.59)	<.001	0.18 (0.05-0.58)	.004
Inadvertent PG excision, No. of PGs	0.20 (0.07-0.57)	<.001	0.19 (0.06-0.54)	.002

Abbreviations: HypoPT, hypoparathyroidism; NIRAF, near-infrared autofluorescence; PG, parathyroid gland; RR, relative risk.

^a^ Crude RR calculated using the χ^2^ test.

^b^ Age-adjusted RR calculated using a multivariate binary regression model.

**Figure 2.**
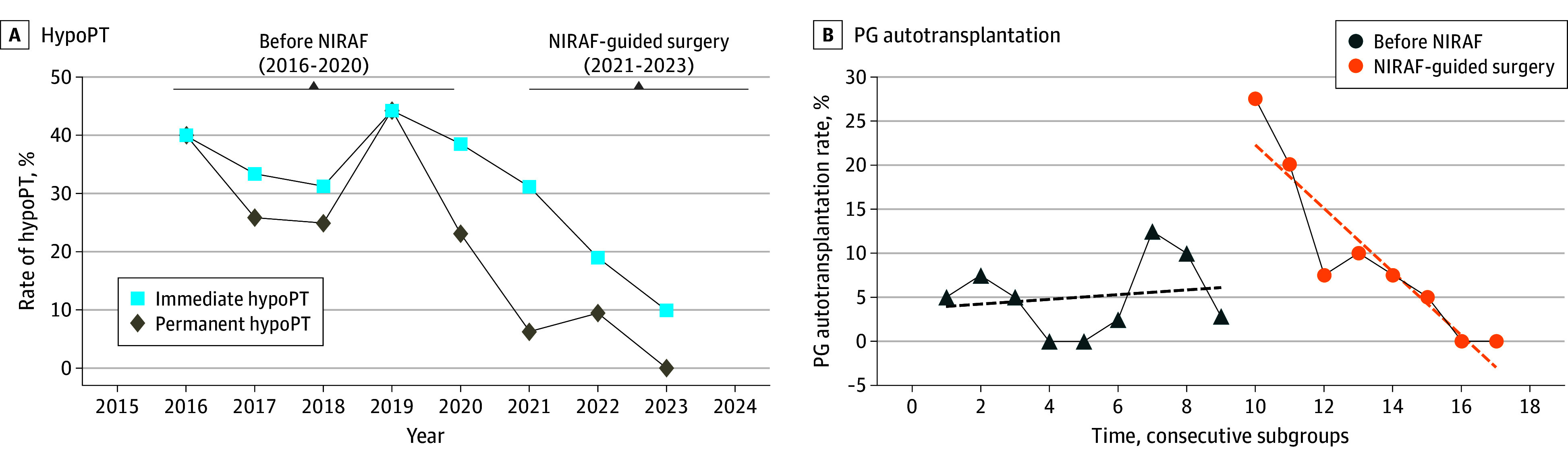
Trends of Hypoparathyroidism (hypoPT) and Parathryoid Gland (PG) Autotransplantation Over Time A, Annual rates of both immediate and permanent hypoPT. B, PG autotransplantation over time. The surgical procedures were divided into subgroups of 10 consecutive total thyroidectomies, and the PG autotransplantation rates were calculated in each subgroup (number of autotransplanted PGs divided by the total number of PGs). A total of 17 subgroups were generated. Of note, subgroups number 9 and number 17 only consisted of 9 and 8 surgical procedures, respectively. By linear regression, a fitted line was created both before and after the introduction of near-infrared autofluorescence (NIRAF). α indicates slope of the fitted line from the linear regression. α(before NIRAF) = 0.27 (95% CI, −1.1 to 1.7; *P* = .66); α(NIRAF) = −3.6 (95% CI, −5.1 to −2.2; *P* = .001).

Figure 2A illustrates the annual rates of both immediate and permanent hypoPT. After the introduction of NIRAF, both rates decreased noticeably over time, reaching 0% for permanent hypoPT at the end of the study.

Parathyroid gland identification rates significantly increased from 61% (95% CI, 56%-66%) to 75% (95% CI, 70%-80%) (*P* < .001) after the introduction of NIRAF (Table 2). Rates of inadvertent PG excision significantly decreased from 21% (95% CI, 13%-31%) to 4% (95% CI, 1%-11%) (*P* = .001) when NIRAF was used, whereas PG autotransplantation occurred more frequently in the NIRAF group (10% [95% CI, 7%-14%]) vs 5% [95% CI, 3%-8%]; *P* = .007). Figure 2B shows that PG autotransplantation rates were high in the NIRAF group in the beginning of the study. Over time, PG autotransplantation rates declined significantly, approximating 0% at the end of the study.

Table 3 shows the crude and age-adjusted RR for all primary and secondary outcomes, comparing NIRAF-guided surgery with conventional surgery. Adjusting for age did not affect the statistical significance for any of the parameters.

NIRAF was used a mean (SD) of 7 (2) times per surgical procedure (Table 2). A total of 148 PGs (63%) were identified with NIRAF before the naked eye. Near-infrared autofluorescence was mostly used on the removed specimen (70 of 78 [90%]), before dissection of the lower thyroid pole (67 of 78 [86%]) and before latero-posterior dissection (61 of 78 [78%]). It was less likely to be used before dissection of the upper thyroid pole (53 of 78 [68%]). Finally, the surgeons stated either some or great benefit of using NIRAF in 73 of 78 cases (94%).

The mean (SD) duration of surgery was higher when NIRAF was used compared with the control group (NIRAF: 137 [30] minutes vs control: 116 [28] minutes; *P* < .001) (Table 2). Patients who underwent NIRAF-guided surgery also had a significantly shorter median (IQR) hospital stay than controls (NIRAF: 2 [1-3] days vs control: 3 [2-4] days; *P* < .001). Biochemically, patients in the NIRAF group had significantly higher levels of both plasma PTH and Ca^2+^ at POD1, first follow-up, and second follow-up. There was no difference in remaining surgery-related and histological outcomes when comparing the 2 groups.

## Discussion

In this cohort study on autofluorescence-guided total thyroidectomy in low-volume, nonparathyroid institutions, we found a significant reduction in both immediate and permanent hypoPT. Before the use of NIRAF, hypoPT rates were critically high, reaching up to 32% for permanent hypoPT. After the introduction of NIRAF, permanent hypoPT rates dropped significantly to 6%, corresponding to an 80% decrease in the risk of permanent hypoPT. Interestingly, hypoPT rates seemed to drop more over time, approximating 0% for permanent hypoPT at the end of the study. This could either indicate a slight modification of the surgical technique over time, or the presence of a potential learning curve related to the use of NIRAF. The pattern of PG autotransplantation when NIRAF was used supports the latter. Autotransplantation rates in the NIRAF group were initially high, but declined significantly over time, also approximating 0% at the end of the study. Thus, the use of NIRAF was not consistent throughout the study period. If NIRAF is not used frequently and early during surgery, PGs will tend to be found on the specimen, resulting in elevated autotransplantation rates. In contrast, the frequent and early use of NIRAF could allow for timely PG identification, hereby reducing autotransplantation rates. Thus, the tendency observed regarding PG autotransplantation rates could possibly explain the tendency observed in the rates of hypoPT over time, both pointing toward the presence of a potential learning curve.

To our knowledge, this is the first study to show a significant reduction in the rate of permanent hypoPT following autofluorescence-guided thyroid surgery. Both Dip et al^27^ and Benmiloud et al^25^ showed a reduction in immediate hypocalcemia, but not in permanent hypocalcemia. In the studies by DiMarco et al,^37^ Lykke et al,^30^ and Bergenfelz et al,^38^ no statistically significant change was reported for either immediate or permanent hypoPT. The overall absence of reduction in the rates of permanent hypoPT in these studies can probably be ascribed to the fact that their permanent hypoPT rates were relatively low. All these studies were conducted at institutions with high surgical volume and/or experience with parathyroid surgery. In comparison, our study took place at low-volume institutions without any experience with parathyroid surgery. In such settings, the rates of permanent hypoPT were found to be very high, reaching up to 32%. The difference in the rates of permanent hypoPT can in part be due to differences in the definitions used. However, differences in the surgical experience may also play a substantial role.^39^ In accordance, Reinke et al^17^ recently showed similar high rates of permanent hypoPT when surgery was performed by surgeons without experience in parathyroid surgery. Thus, altogether, these findings suggest the presence of a potential gap between the thyroid-only surgeon and the parathyroid surgeon in terms of adequate PG preservation.^13^ From an objective point of view, it does not seem unreasonable to assume that parathyroid surgeons may have better prerequisites for PG identification, localization, and management than thyroid-only surgeons. Interestingly, our findings suggest that NIRAF potentially holds the ability to reduce that gap between the thyroid-only surgeon and the parathyroid surgeon, as we saw a remarkable drop in hypoPT rates following NIRAF-guided surgery. In other words, the use of NIRAF may be particularly useful for low-volume, nonparathyroid surgeons, as it seems to make their hypoPT rates comparable with experienced parathyroid surgeons, which indeed is an interesting finding.

It is noticed that hypoPT rates seemed to decline the last year prior to the introduction of NIRAF. In general, hypoPT rates were fluctuating before the introduction of NIRAF, and the drop observed in 2020 was most likely a reflection of the very high hypoPT rates observed in 2019. Furthermore, the hypoPT rates observed in 2020 were similar to those observed in 2017 and 2018. It is therefore unlikely that the overall reduction in hypoPT observed after the introduction of NIRAF had commenced before the introduction of NIRAF.

More PGs were identified when NIRAF was used in our study, and less PGs were accidentally removed. Both factors could contribute to the decreased rates of hypoPT found. This is in line with what was reported by Benmiloud et al.^25^ However, even with NIRAF, 25% of theoretically present PGs were not identified in our study. In part, this could be explained by the presence of intrathyroidal PGs and/or deeply located PGs covered with substantial fat tissue. Such situations would hinder optimal NIRAF visualization, as near-infrared penetration range is only a few millimeters.^40^ To expect that NIRAF by itself can enable the visualization of all PGs in every case is therefore unrealistic.

Interestingly, and in accordance with Benmiloud et al,^25^ 63% of PGs identified in our study were identified with NIRAF before the naked eye. This is of particular interest, as early PG identification increases the attention toward PG locations before extensive dissection has been performed, hereby increasing the chance of preserving functional PGs. The increased duration of surgery seen in the NIRAF group may be a reflection of these findings. As more PGs were found in NIRAF-guided surgery, in addition to the fact that PGs were found early in the process, more time would most likely have been spent in dissecting areas adjacent to the locations of PG. The same tendency of an increased duration of surgery was also reported by Benmiloud et al.^25^

Near-infrared autofluorescence was mostly used on the removed specimen, before dissection of the lower thyroid pole and before latero-posterior dissection. Application of NIRAF prior to dissection of the upper thyroid pole was less common. Considering the variability of PG locations, it is not unlikely that an even more systematic use of NIRAF could have yielded even lower rates of hypoPT. Finally, the surgeons reported experiencing either some or great benefit from using NIRAF in 94% of the cases, which indeed is a high satisfaction rate.

### Limitations

This study had limitations. Patients in the NIRAF group were a few years older than controls, hence the 2 groups were not completely comparable at baseline. This must be considered a limitation of our study, as age in theory could be a confounder. However, a thorough adjustment for age was made for all our primary and secondary outcomes. Adjusting for age did not alter the associations found and the statistical significance remained intact. Thus, it is unlikely that age has confounded our results. Another main limitation of our study is the before-and-after design. In such design, the Hawthorne effect will always be present,^25^ because it is methodologically impossible to determine whether the observed results are caused by the use of NIRAF or an increased surgical attention. Randomized clinical trials are less prone to be affected by the Hawthorne effect and could therefore with benefit be performed to further enlighten the associations found in our study. However, the thorough and detailed evaluation of the use of NIRAF in our study has revealed numerous associations that all may point toward a potential causality.

## Conclusions

This study found that rates of both immediate and permanent hypoPT following total thyroidectomy decreased significantly after the introduction of NIRAF in low-volume, nonparathyroid institutions. These findings suggest that near-infrared autofluorescence may help reduce a potential gap between the low-volume, thyroid-only surgeon and the experienced parathyroid surgeon, making their hypoPT rates comparable. Finally, our findings also suggest the presence of a learning curve related to the use of NIRAF.

## Notes

Supplement.
Data Sharing Statement
