# Supplementary material for: Autofluorescence-Guided Total Thyroidectomy in Low-Volume, Nonparathyroid Institutions
Source: JAMA Netw Open. 2024 May 15;7(5):e2411384. doi: 10.1001/jamanetworkopen.2024.11384 (PMC11096987; doi:10.1001/jamanetworkopen.2024.11384)
Supplement: Supplement. — Data Sharing Statement [file jamanetwopen-e2411384-s001.pdf]

Data Sharing Statement

Abood. Autofluorescence-Guided Total Thyroidectomy in Low-Volume, Nonparathyroid Institutions. *JAMA Netw Open*. Published May 15, 2024.
doi:10.1001/jamanetworkopen.2024.11384

Data
Data available: No
